# Biological Measures in the WLS: Genetic and Microbiome Data

**DOI:** 10.1093/geroni/igab046.853

**Published:** 2021-12-17

**Authors:** Kamil Sicinski

**Affiliations:** University of Wisconsin-Madison, Madison, Wisconsin, United States

## Abstract

Ever since releasing genotype data in 2017, the WLS continually expands resources available to users interested in genetic research. Key advantages to the WLS data for genetics research include its sibling sample and nearly full life course longitudinal study design. In 2021, we now have state-of-the-art polygenic scores available in multiple domains, such as health, cognition, fertility, personality, risk behaviors and attitudes, and life satisfaction. The scores cover phenotypes spanning from adventurousness, through educational attainment, to age at which voice deepened. Additionally, the genotype data was re-imputed in 2021 to the superior Haplotype Reference Consortium reference panel and the WLS expects to obtain copy number variants data next year. In addition to genetic data, we have a set of novel microbiome data on a subset of participants that allows researchers to study relationships between environments and gut microbial composition.

